# Editorial: A digitally-enabled, science-based global pandemic preparedness and response scheme: how ready are we for the next pandemic?

**DOI:** 10.3389/fpubh.2024.1429615

**Published:** 2024-06-14

**Authors:** Yi-Roe Tan, Brian Li Han Wong, Sylvia Kiwuwa-Muyingo, Serge Stinckwich, Peiling Yap

**Affiliations:** ^1^HealthAI, Geneva, Switzerland; ^2^Department of International Health, Care and Public Health Research Institute, Maastricht University, Maastricht, Netherlands; ^3^Center for Resilient Health, Stockholm School of Economics (SSE), Stockholm, Sweden; ^4^Department of Global Public Health, Karolinska Institute, Stockholm, Sweden; ^5^Digital Health Section, European Public Health Association (EUPHA), Utrecht, Netherlands; ^6^Digital Public Health Task Force, Association of Schools of Public Health in the European Region (ASPHER), Brussels, Belgium; ^7^African Population and Health Research Center, Nairobi, Kenya; ^8^United Nations University Institute in Macau, Macau, Macau SAR, China

**Keywords:** digital health, pandemic preparedness and response, public health, digital transformation, artificial intelligence, health systems strengthening, COVID-19, digital health governance

The COVID-19 pandemic has demonstrated how a lack of accurate, real-time outbreak data and an inconsistent science-based response framework can lead to global struggles in responding to the pandemic in a timely and effective manner (1, 2). There is a need to fundamentally transform the international pandemic surveillance and response system, as called for by the World Health Organization (3). To achieve this transformation, scientists and experts worldwide must co-develop a global pandemic preparedness and response scheme that is science-based, digitally-enabled, and works across the continuum of pandemic phases, namely preparedness, surveillance, response, and recovery. In addition, capacity development efforts and robust governance frameworks are crucial in facilitating a sustainable digital transformation for health systems (4, 5). This global pandemic scheme must be built collaboratively and transparently with international organizations, academia, private sector, civil society, and citizens for it to be a trusted source of information for public health decision-making.

The International Digital Health and AI research collaborative (I-DAIR), now known as HealthAI—The Global Agency for Responsible AI in Health, together with its multi-disciplinary panel of scientific experts, developed a research and development (R&D) agenda to build out an end-to-end scheme for pandemic preparedness and response (6). The R&D agenda highlighted four priority areas across the continuum of data generation-modeling-visualization, including (i) Discovering unusual and diverse data sources as well as building population cohorts for equitable data curation; (ii) Building models that could be Findable, Accessible, Interoperable, and Reusable (FAIR) and which would allow for citizen inputs through participatory approaches; (iii) Designing visualizations that are targeted for different stakeholders as well as developing effective communication interfaces between researchers, governments, and citizens; (iv) Cross-cutting issues such as the governance of data and digital technologies, including AI, as well as human and infrastructure capacity development efforts.

A crucial concept connecting these four priority areas is strong citizen engagement. From past outbreaks, it is evident that trust-building among stakeholders who are interdependent in their response to an outbreak resulted in a more effective response (7, 8). To build trust and increase public compliance and effectiveness of mitigation strategies, we need to recognize the role of communities in all phases of the pandemic continuum and across the continuum of participatory data generation-modeling-visualization (9–12). This Research Topic sets out to understand the current state-of-the-art in the four R&D areas, emphasizing citizen engagement enabled by digital means. We received a wide range of submissions covering various approaches and methodologies, demonstrating the multifaceted nature of pandemic preparedness and response. The submissions which have been accepted so far include original research and conceptual analysis articles.

For instance, Mondal et al. explored the use of Twitter as a crowdsourcing platform to gather public opinion on measures to hasten the end of the COVID-19 pandemic, an approach which underscores the potential of social media in engaging a global audience for rapid idea generation at the community level. Similarly, Ogbuokiri et al. utilized clustered geo-tagged Twitter posts to inform and better analyze city-level variations in sentiments toward COVID-19 vaccine-related topics in the three largest South African cities. Both studies highlighted the utility and potential of non-traditional data sources and platforms for gathering citizen insights to shape decision-making and health policy planning in pandemic preparedness and response.

Fournier-Tombs presented the Transplantation, Adaptation and Creation (TAC) framework applied in the public health context, notably to models used during the COVID-19 pandemic. This method of assessing the localization of different elements of an AI system can help guide AI for public health developers and public health officials in conceptualizing model localization. Thinking locally at all stages of the AI development lifecycle aligns well with inclusive models such as Arnstein's ladder for citizen participation, which examines citizen agency in projects (13). Similarly, AI model localization is about the autonomy and empowerment of those who are affected by the technologies, from conceptualization, to development, to governance and use.

Kiwuwa-Muyingo et al. identified a gap in standardized mechanisms for collecting, documenting, and disseminating COVID-19-related data or metadata, which presents challenges in data use and reuse. The study examined the use of the Observational Medical Outcomes Partnership (OMOP) as the Common Data Model (CDM) in the cloud as a Platform as a Service (PaaS) for COVID-19 data. Individual research institutions can utilize the PaaS for accessing FAIR data management, analysis, and sharing capabilities, with data-sharing agreements allowing data producers to retain control over their data. Bhattacharjee et al. further outlined the implementation process for creating a data hub to harmonize population health data from diverse sources in the African region into OMOP CDM. Both studies underscored the need for robust data governance and sharing mechanisms in digital technologies, vital for fostering citizen participation and generating actionable insights to inform policy and decision-making.

Zhao et al. evaluated the anti-pandemic resilience of countries along the Belt and Road route. Countries were classified into different resilience levels through hierarchical clustering, using institutional, infrastructural, economic, social, and technological resilience as components of overall anti-pandemic resilience. Countries with high resilience demonstrated better institutional and economic resilience, whereas countries with low resilience lagged in both infrastructural and social resilience. This suggests a gap and disparity in capacity development efforts across different countries, which must be addressed to build an equitable global pandemic preparedness and response scheme.

In conclusion, the insights gained from these R&D areas and their interlinkages provide a solid foundation for building out a science-based, digitally-enabled, end-to-end global pandemic preparedness and response scheme. The integration of citizen engagement at all points is crucial for building trust and enhancing the effectiveness of pandemic management strategies. As we forge ahead, further research in these areas is vital, not only to solidify our current understanding but also to open new avenues for innovation and exploration in global health resilience.

## Author contributions

Y-RT: Conceptualization, Writing – original draft. BW: Conceptualization, Writing – review & editing. SK-M: Writing – review & editing. SS: Writing – review & editing. PY: Conceptualization, Supervision, Writing – review & editing.
